# Supplementary material for: Pilot Proteomic Analysis of Urinary Extracellular Vesicles Supports the “Toxic Urine Hypothesis” as a Vicious Cycle in Refractory IC/BPS Pathogenesis
Source: Int J Mol Sci. 2025 Dec 22;27(1):130. doi: 10.3390/ijms27010130 (PMC12786074; doi:10.3390/ijms27010130)
Supplement: Supplementary file 1 [file ijms-27-00130-s001.zip › ijms-4024719-supplement_1218.pdf]

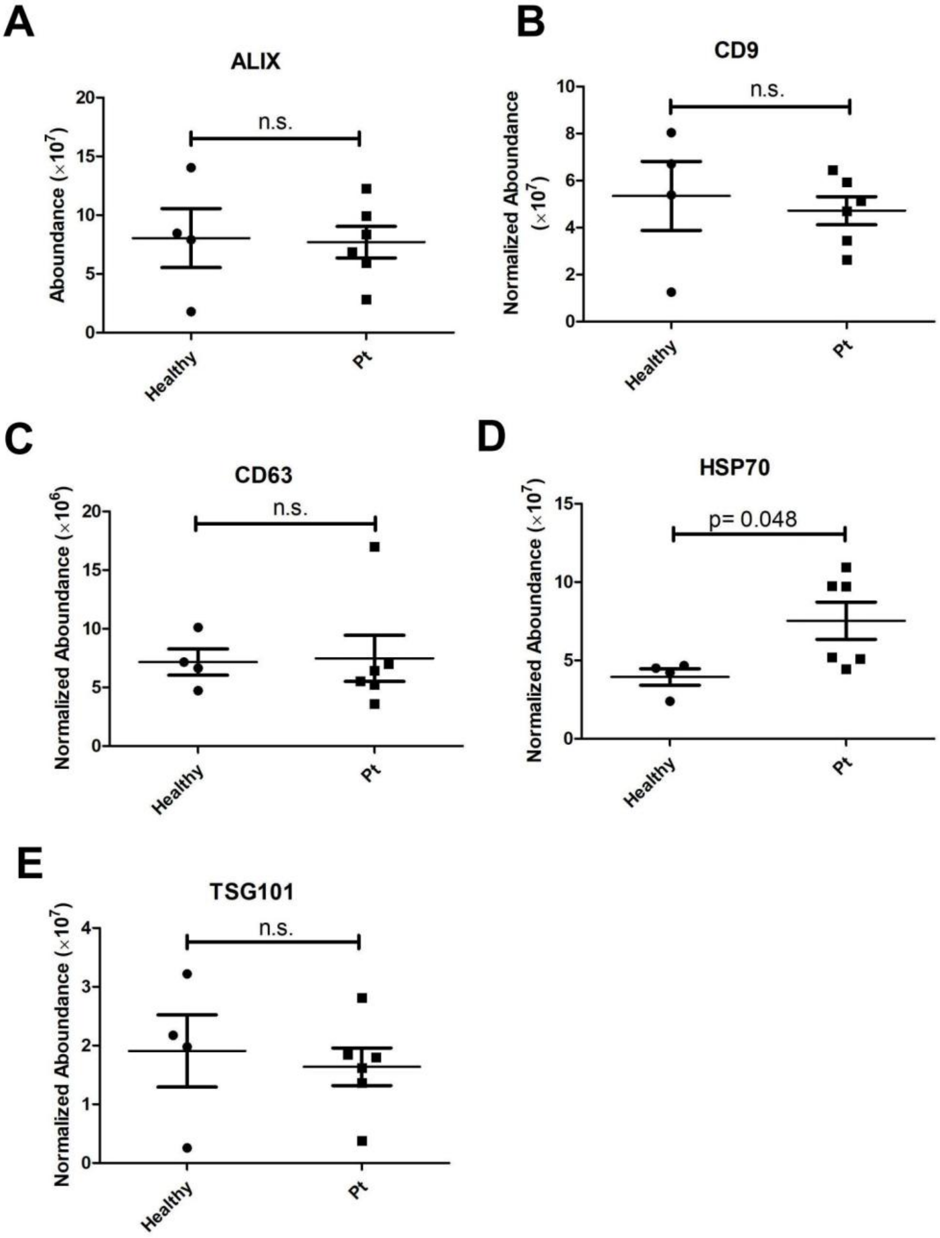

Figure S1. Abundance of common extracellular vesicle markers in urinary extracellular vesicles (uEVs). Normalized abundances of ALIX (A), CD9 (B), CD63 (C), HSP70 (D), and TSG101 (E) in uEVs isolated from healthy donors (Healthy) and patients with IC/BPS (Pt) are shown as mean ± SEM. Group comparisons were analyzed using an unpaired two-tailed Student's *t* test. n.s., not significant.

Table S1. Antibodies used in this study

Product	Source	Catalog No.
**Primary antibody**		
Alix	Proteintech Group, Inc	12422-1-AP
HSP70	Enzo Life Sciences	ADI-SPA-810-488
CD9	Proteintech Group, Inc	60232-1-lg
Tsg101	Santa Cruz Biotechnology	sc-7964
GAPDH	GeneTex International Corporation	GTX100118
14-3-3ζ	Servicebio	GB111023
NF-κB p65	Cell Signaling Technology	#8242
p-NF-κB p65(ser536)	Cell Signaling Technology	#3033
**Secondary antibodies**		
anti-rabbit IgG-HRP	GeneTex International Corporation	GTX21311201
anti-mouse IgG-HRP	GeneTex International Corporation	GTX213111-01
