# Supplementary figures and images for: Pilot Proteomic Analysis of Urinary Extracellular Vesicles Supports the “Toxic Urine Hypothesis” as a Vicious Cycle in Refractory IC/BPS Pathogenesis
Source: Int J Mol Sci. 2025 Dec 22;27(1):130. doi: 10.3390/ijms27010130 (PMC12786074; doi:10.3390/ijms27010130)

Fig. 1E

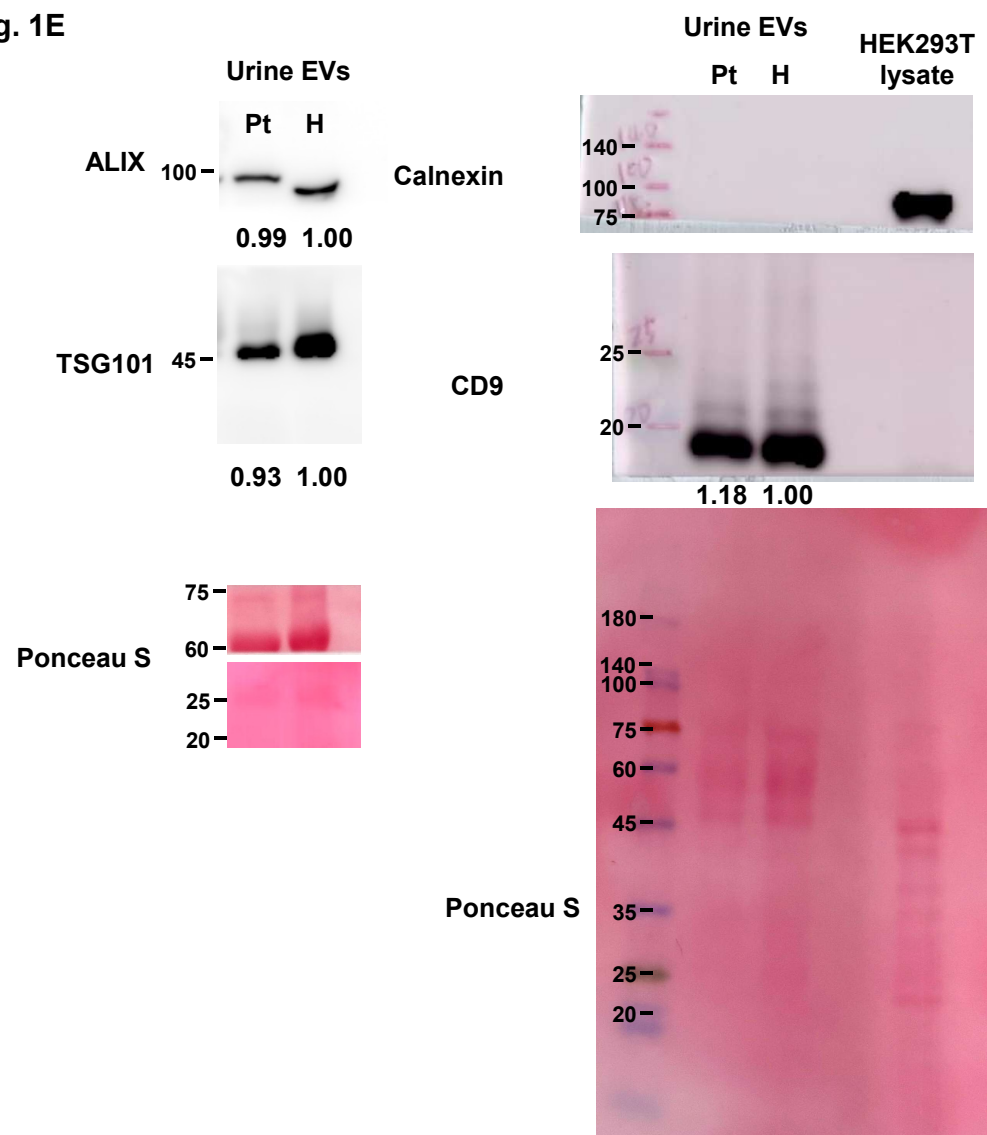

Fig. 5C

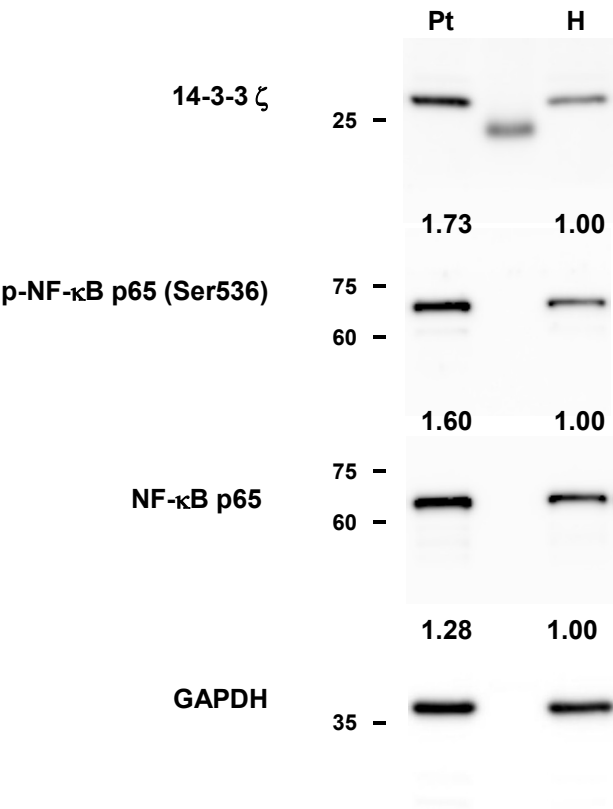

Supplement: Supplementary file 1 [file ijms-27-00130-s001.zip › WB full blot images_R1.pdf]
